# Integration of operator-validated contours in deformable image registration for dose accumulation in radiotherapy

**DOI:** 10.1016/j.phro.2023.100483

**Published:** 2023-08-20

**Authors:** Lando S Bosma, Mario Ries, Baudouin Denis de Senneville, Bas W Raaymakers, Cornel Zachiu

**Affiliations:** a Department of Radiotherapy, UMC Utrecht, Heidelberglaan 100, 3508 GA Utrecht, The Netherlands; b Institut de Mathématiques de Bordeaux (IMB), UMR 5251 CNRS/University of Bordeaux, F-33400 Talence, France; c Imaging Division, UMC Utrecht, Heidelberglaan 100, 3508 GA Utrecht, The Netherlands

**Keywords:** Contour guidance, Deformable image registration, Deformable dose warping, Adaptive radiotherapy, Constrained motion estimation, Preconditioning

## Abstract

**Background and Purpose:**

Deformable image registration (DIR) is a core element of adaptive radiotherapy workflows, integrating daily contour propagation and/or dose accumulation in their design. Propagated contours are usually manually validated and may be edited, thereby locally invalidating the registration result. This means the registration cannot be used for dose accumulation. In this study we proposed and evaluated a novel multi-modal DIR algorithm that incorporated contour information to guide the registration. This integrates operator-validated contours with the estimated deformation vector field and warped dose.

**Materials and Methods:**

The proposed algorithm consisted of both a normalized gradient field-based data-fidelity term on the images and an optical flow data-fidelity term on the contours. The Helmholtz-Hodge decomposition was incorporated to ensure anatomically plausible deformations. The algorithm was validated for same- and cross-contrast Magnetic Resonance (MR) image registrations, Computed Tomography (CT) registrations, and CT-to-MR registrations for different anatomies, all based on challenging clinical situations. The contour-correspondence, anatomical fidelity, registration error, and dose warping error were evaluated.

**Results:**

The proposed contour-guided algorithm considerably and significantly increased contour overlap, decreasing the mean distance to agreement by a factor of 1.3 to 13.7, compared to the best algorithm without contour-guidance. Importantly, the registration error and dose warping error decreased significantly, by a factor of 1.2 to 2.0.

**Conclusions:**

Our contour-guided algorithm ensured that the deformation vector field and warped quantitative information were consistent with the operator-validated contours. This provides a feasible semi-automatic strategy for spatially correct warping of quantitative information even in difficult and artefacted cases.

## Highlights

• We introduce a contour-guided registration method for enhanced anatomical fidelity.
• The method was multi-modal, fast and robust over multiple anatomies and deformations.
• Our algorithm performed significantly better compared to algorithms without guidance.
• It decreased the voxelwise registration and dose warping error by factors of 1.2-2.0.
• The method incorporated operator-validated contours into dose warping applications.

## 1 Introduction

Deformable image registration (DIR) plays an important role in image-guided adaptive radiotherapy. Currently, it is widely used for contour propagation, warping the planning contours to the anatomy of the day. The application of DIR for warping and/or accumulating quantitative information such as radiation dose or Hounsfield units is increasing [1], [2], [3]. In clinical workflows, the contours generated by DIR undergo visual inspection by an operator and may be adjusted. Thereby the underlying estimated deformation becomes locally invalid and in turn, the warping of quantitative information is inconsistent. A key challenge in incorporating automatic DIR into clinical workflows that involve warping quantitative information is to provide a suitable hands-on repair strategy for this scenario. Indeed, recent surveys of radiotherapy centers found that an important barrier to the clinical adoption and use of DIR was to determine what to do when a registration is unsatisfactory [4], [5]. On the other hand, due to this workflow, every daily image-guided adaptive radiotherapy treatment fraction has these operator-approved contours available.

Contours have been previously used to guide image registration. Gu and colleagues proposed a contour-guided adaption of the image intensity-based demons algorithm[6]. An additional term in the demons cost function matches the intensities of modified images constructed by incorporating one or multiple contour pair(s) onto the original images. This method has a high memory demand as it requires a new set of images for every contour used for guidance, and it is sensitive to the tuning of multiple free parameters. This algorithm was also suitable for mono-modal image registrations, which can become a limitation. Multi-modal image registration is important for image-guided radiotherapy as it allows to combine modality-specific information from Computed Tomography (CT) and multi-contrast Magnetic Resonance (MR) images in the same reference frame. Multi-modal deformable image registration remains a particularly challenging task for state-of-the-art DIR algorithms. Recently, contours were used to segment part of the images to consider for registration, resulting in a transformation per organ that was validated for dose warping [7]. Alam and colleagues used an algorithm that optimized both image similarity and structure guidance [8]. The algorithm was shown to improve contour overlap compared to rigid registration and subsequently applied to dose accumulation. In other work, a multi-modal contour-guided algorithm was shown to improve contour-propagation [9]. The algorithm was however slower, at about 15 min per registration. A commercial registration solution exists that can combine the matching of image similarities with a minimization of contour surface distances [10].

The adoption of deep learning segmentation in the clinic is increasing [11], [12], [13]. These automatically generated contours can also be used as input for registration methods (after manual validation). In that way, this information can be used for the contour-propagation of structures that are not segmented and for warping quantitative information in accordance with these structures.

The aims of this paper are as follows: develop a solution for the integration of operator-validated or corrected contours into the registration for consistent dose warping and/or accumulation; design this method to be compatible with the low latency required by online workflows, as well as suitable for use in the scope of multi-modal applications; validate the algorithm for multiple anatomies, deformation patterns, and image modalities using multiple benchmarks relevant to adaptive image-guided radiotherapy; and explicitly test its application to the warping of quantitative information such as dose and/or Hounsfield units.

## 2 Materials and methods

### 2.1 Proposed registration algorithm

To incorporate contour information in the deformable image registration process, we combined the image data fidelity term D and regularization term R of EVolution [14] with an optical flow data fidelity term on the binary masks of the contours [15]:

(1) $$\begin{array}{cc} E_{\mathrm{CG}} & =\int_{\Omega }D_{\mathrm{images}}(I_{r},I_{m},\overset{\to }{u})+\beta \cdot D_{\mathrm{contours}}(C_{r},C_{m},\overset{\to }{u})+\alpha \cdot R_{\mathrm{smoothness}}(\overset{\to }{u})\\ & =\int_{\Omega }\exp(f(\overset{\to }{u},I_{r},I_{m}))+\beta {\left(\nabla C_{m}\cdot \overset{\to }{u}+C_{m}-C_{r}\right)}^{2}+\alpha \left(||\overset{\to }{\nabla }u_{1}|{|}_{2}^{2}+||\overset{\to }{\nabla }u_{2}|{|}_{2}^{2}+||\overset{\to }{\nabla }u_{3}|{|}_{2}^{2}\right), \end{array}$$

with

(2) $$f(\overset{\to }{u}(\overset{\to }{r}),I_{r},I_{m}))=-\frac{\underset{\overset{\to }{s}\in \Gamma (\overset{\to }{r})}{\sum }\left|\overset{\to }{\nabla }I_{r}(\overset{\to }{s})\cdot \overset{\to }{\nabla }I_{m}(\overset{\to }{s}+\overset{\to }{u}(\overset{\to }{s}))\right|}{\underset{\overset{\to }{s}\in \Gamma (\overset{\to }{r})}{\sum }\|\overset{\to }{\nabla }I_{r}(\overset{\to }{s}){\|}_{2}\|\overset{\to }{\nabla }I_{m}(\overset{\to }{s}+\overset{\to }{u}(\overset{\to }{s})){\|}_{2}},$$

where u is the deformation vector field with components $u_{1,2,3},I_{r,m}$ are the reference and moving images, $C_{r,m}$ the reference and moving contours, and $\Gamma (\overset{\to }{r})$ is a neighborhood around $\overset{\to }{r}$. There are two free parameters weighting the contour guidance (β) and regularization (α). The performance of the algorithm was investigated for α∈[0.4,1.2],β∈[0.5,2.5] while α=1.0 and β=2.0 were used for all experiments in this manuscript.

We used an iterative fixed-point scheme on the Euler–Lagrange equations derived from Eq. 1. Their derivations are given in Supplementary Material A. The registration was performed using a coarse-to-fine scheme, starting the iterations on the 16-fold downsampled images and contours, and upsampling with factors of two. We used iterative refinement, restarting the registration process 50 times at each resolution level. Each iteration was stopped when the average variation of the motion magnitude from one update to the next was smaller than $10^{-3}$ voxels. The deformations from the previous refinement iteration were then used as a starting point [16].

The algorithm was implemented using the Compute Unified Device Architecture (CUDA) and executed on a Nvidia Quadro RTX 5000 graphics card.

### 2.2 Helmholtz-Hodge decomposition

Using contour-guidance may introduce the risk of over-constraining, leading to anatomically implausible deformations. Therefore, we introduced the Helmholtz-Hodge decomposition as an optional post-processing step [17], [18], [19]. This was used to decompose the estimated deformation vector field into three components: a curl-free component, a divergence-free component, and a harmonic remainder that is both curl-free and divergence-free. The details of its derivation and computation are presented in Supplementary Material B. The Helmholtz-Hodge decomposition thus provided local control over the registration result and allowed to demand incompressible (i.e. divergence-free) deformations in incompressible regions, to potentially resolve the risk of over-fitting.

### 2.3 Test data and evaluation methods

We tested our algorithm on experiments representing misregistrations of different origins. These experiments will be discussed in detail below. An overview of the anatomies, modalities, and evaluation criteria used for the experiments can be found in Table 1. For all datasets, we evaluated the contour correspondence using the mean distance to agreement and the Hausdorff distance [20] and the anatomical plausibility using the range of the Jacobian determinant on incompressible organs. For the simulated datasets, we evaluated the voxelwise endpoint error [21], i.e. the Euclidean distance between the benchmark and estimated vector for each voxel, and dose warping error. Additional details of the evaluation criteria and acquisition parameters used are provided in Supplementary Material C and D.

**Table 1:** Overview of the test data used, with the experiment name indicating its relevance, the organ contour(s) used for guidance and evaluation of contour correspondence, the modalities and image types involved, and the evaluation criteria used. Evaluation criteria were the Hausdorff distance (HDD), Jacobian determinant (JD, evaluated on the indicated contour), target registration error (TRE), endpoint error (EE), and dose warping error (DE).

Experiment name	Contours	Modalities	Evaluation	
Large complex deformations	Prostate	3D T2w MRI	HDD, JD	
Large complex deformations	Lungs	3D CT	HDD, JD, TRE	
Signal dropout	Prostate	3D cine MRI	HDD, JD	
Signal dropout simulation	Prostate	3D cine MRI	HDD, JD, EE, DE	
Multi-modal	Liver, spleen, kidneys	3D CT & 3D T1w MRI	HDD, JD	
Cross-contrast simulation	Prostate	3D DIXON MRI	HDD, JD, EE	

Our proposed contour-guidance algorithm was compared to the original EVolution implementation 1 and to the mutual-information B-spline algorithm from the openly available Elastix toolbox [22], [23]. Details on the parameters used are given in Supplementary Material E. We compared the results both with and without the Helmholtz-Hodge decomposition. We performed statistical testing using the paired t-test.

*Large and complex deformations datasets.* Using cone-beam CT linac systems [24] or the MR-linac [25], [26], [27], treatment plans can be updated to the anatomy of the day. Image registration can be used to propagate the contours to the new anatomy, and to perform dose accumulation. This can be challenging when large day-to-day anatomical variations occur. We used pretreatment (T2w) MR and daily MR scans for 20 prostate cancer patients (5x7.25 Gy) with delineations of the bladder, prostate and rectum on both image sets made by experienced radiation oncologists. Ethical approval for use of all internally acquired patient data was provided by the Ethics Board of the University Medical Center Utrecht.

Registration of thoracic inhale to exhale images represents a challenge for image registration due to the large magnitude of the deformations as well as their complex nature at the lung-liver interface and the sliding motion between the lungs and the ribs. We tested our algorithm on twenty thoracic 4DCT image pairs from the DIR-lab and COPD-gene datasets^2^ [28], [29]. For images of full inhale and full exhale, 300 manually annotated anatomical landmarks were available to quantify the target registration error. An experienced staff member delineated the lung contours on both image sets.

*Signal dropout datasets.* With the MR-linac, the patient’s anatomy can be imaged during treatment. This can be used to track the tumor and to reconstruct the delivered dose. Typically, this is done with bSSFP-sequences that offer sufficient anatomical detail for organ tracking combined with low acquisition and reconstruction times. The problem is that these sequences are prone to susceptibility artefacts, caused e.g. by gas pockets in the rectum.

We tested the algorithm on a 4D cine-MR series acquired during treatment of a prostate cancer patient on the 1.5T MR-Linac Unity system (Elekta AB, Stockholm, Sweden) installed at the UMC Utrecht, The Netherlands. During imaging, a signal dropout appeared due to a gas bubble passing through the rectum, see Figure S1 in the Supplementary Material.

To quantify the accuracy of the resulting deformation vector field, we also simulated a cine-MR with a synthetic signal dropout for a prostate cancer patient. First, we simulated a clinically observed and anatomically plausible rectal filling organ movement [30] using the biomechanical modeling software FEBio [31]. Thereafter an artificial signal dropout was created on the moving image, see Figure S1 in the Supplementary Material. The mean planned dose on the prostate for this patient was 62.6 Gy.

*Multi-modal and cross-contrast datasets.* CT-to-MR registration is needed in radiotherapy to combine information from both of these modalities. Especially for adaptive radiotherapy on the MR-Linac, it is essential to warp the electron density or planned dose distribution from the planning CT to the MR of the anatomy of the day. In the lower abdomen, a lot of anatomical changes can happen that make for a challenging registration task that in turn may lead to corrections in the propagated contours. We used abdominal CT and MR scans for 8 patients from the Learn2Reg challenge^3^ [32]. The data was selected from The Cancer Imaging Archive project [33], [34], [35], [36] and manual segmentations of the liver, spleen, right kidney and left kidney were added by the organizers. We have cropped the images for a matching field of view.

To quantify the accuracy of the resulting deformation vector field, we also simulated a cross-contrast experiment using a set of DIXON images of a prostate cancer patient. These images were acquired in the same anatomical state, allowing the simulation of the deformation of one of the images with a known benchmark. A typically observed prostate deformation was simulated using biomechanical modeling software FEBio, which resulted in the prostate moving in the anterior and caudal direction. The in-phase image was deformed to create the moving image and the water-only image was used as the reference image.

## 3 Results

**Fig. 1:**
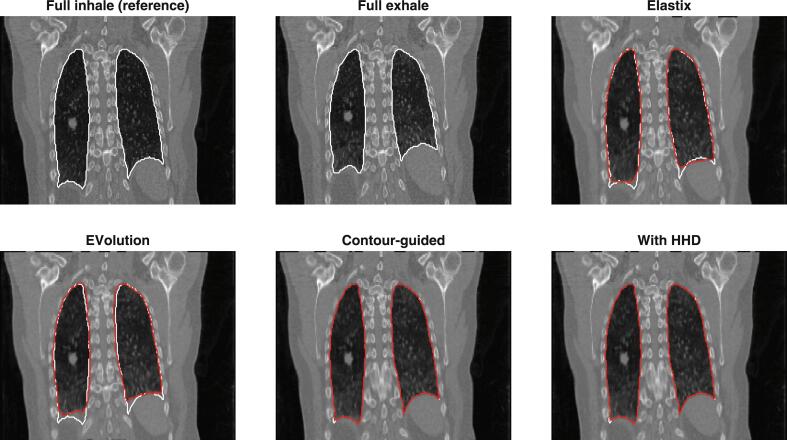
An example case for the experiment on large complex deformations of the thorax with CT-to-CT registrations. A coronal slice of the full inhale and full exhale images is shown (TRE before registration 10.9 mm), as well as the exhale image registered to the inhale using Elastix (TRE 2.9 mm), the original EVolution (3.9), our proposed contour-guided algorithm (1.7), and this contour-guided algorithm with the Helmholtz-Hodge decomposition (HHD) on the body excluding the lungs (1.8). The lung contours used for guidance are shown in white and the registered contours are shown in red. In particular, the caudal side of the lungs is better aligned when using contour-guidance.

A visual comparison of a thorax CT-to-CT registration with and without contour-guidance demonstrated that in particular the caudal boundary of the lungs matched better when using contour-guidance, see Fig. 1. Also for MR-to-MR and MR-to-CT registrations, an improved contour and image overlap was visible, see Figures S2 and S3 in the Supplementary Material. For all three experiments, the case with results closest to the mean of the dataset is shown.

The proposed algorithm was relatively stable with respect to the free parameters α and β, see Figures S4, S5, and S6 in the Supplementary Material. The difference in error between the used configuration and the optimal one was low at 6 to 8%.

The GPU-accelerated EVolution and GPU-accelerated contour-guided EVolution were considerable faster than Elastix, see Table S3 in the Supplementary Material. Using contour-guidance decreased the registration time for the prostate and abdomen anatomies, but increased the time for the thorax anatomies.

### 3.1 Contour correspondence and anatomical plausibility

The mean distance to agreement decreased by a factor of 1.9 on average by using contour-guidance, see Table 2. After the Helmholtz-Hodge decomposition the contour overlap was still considerably improved. For the Hausdorff distance, qualitatively similar results were found, see Table S1 in the Supplementary Material.

**Table 2:** Mean distance to agreement in mm for the different experiments when using no registration, Elastix, EVolution without contour-guidance, the proposed algorithm with contour-guidance, and the proposed algorithm with contour-guidance and the Helmholtz-Hodge decomposition (HHD). For the experiments with multiple registrations the mean (standard deviation) is shown. Contour-guidance reduced the distance by a factor of 7.0 on average (range 1.3–13.7), compared to the best algorithm without guidance. This was statistically significant for all experiments (p<0.01). The contour overlap after the HHD was still significantly (p<0.03) improved. The mean distance to agreement split per organ for the abdomen experiment is shown in Table S2 in the Supplementary Material.

Experiment	No DIR (mm)	Elastix (mm)	EVolution (mm)	Contour-guided (mm)	With HHD (mm)		
Large complex deformations prostate	9.8 (12.1)	1.1 (1.1)	0.8 (1.0)	0.1 (0.2)	0.2 (0.2)		
Large complex deformations thorax	2.0 (2.2)	0.1 (0.0)	0.1 (0.1)	0.0 (0.0)	0.1 (0.0)		
Signal dropout prostate	1.0	0.7	0.4	0.0	0.1		
Signal dropout simulation prostate	0.6	0.1	0.1	0.1	0.1		
Multi-modal abdomen	13.3 (12.0)	6.0 (12.5)	4.6 (9.3)	0.7 (2.4)	1.8 (2.9)		
Dixon cross-contrast simulation prostate	7.9	0.6	0.4	0.1	0.1		

The Helmholtz-Hodge decomposition decreased the non-outlier range of the Jacobian determinant by a factor of 2.0 on average, see Table S3 in the Supplementary Material. It also brought the values closer to the benchmark ranges for the biomechanical simulations. The decomposition furthermore resolved any undesired negative (outlier) values that indicate the estimation of tissue folding.

### 3.2 Registration errors and dose warping errors

**Fig. 2:**
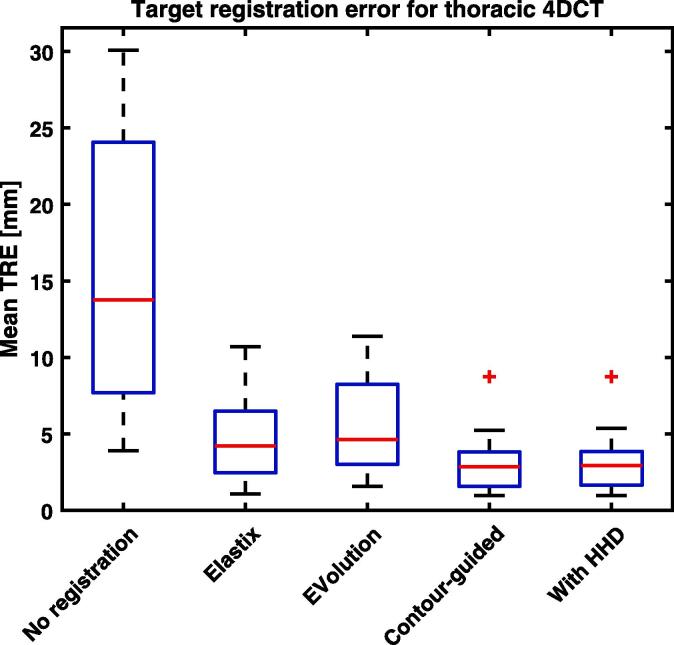
Box plot of the mean target registration error (TRE) for the large complex deformations of the thorax CT-to-CT when using no registration, Elastix, EVolution without contour-guidance, the proposed algorithm with contour-guidance, and the proposed algorithm with contour-guidance and the Helmholtz-Hodge decomposition (HHD). Contour-guidance on the lungs significantly ($p<10^{-4}$) decreases the mean error compared to registration without guidance for all cases, on average by a factor of 1.3 and 1.8. The error after performing a Helmholtz-Hodge decomposition (HHD) is very similar.

For the manually annotated 4DCT, the mean target registration error over the 20 cases was 15.9 mm before registration, see Fig. 2. Using Elastix and EVolution this became 4.3 and 5.6 mm. Including contour-guidance decreased the error by a factor of 1.3 and 1.8, to 3.2 mm. Applying the Helmholtz-Hodge decomposition kept the error at 3.2 mm.

**Fig. 3:**
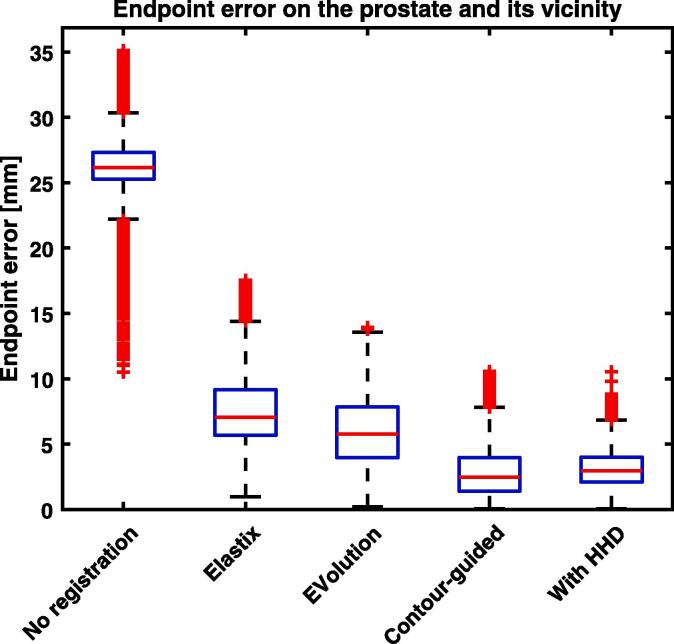
Box plot of the endpoint error on the prostate and its vicinity of 2 mm for the cross-contrast biomechanical simulation of a prostate MRI. Shown are the results without registration, using Elastix, using EVolution without contour-guidance, our algorithm with contour guidance, and the algorithm with contour-guidance combined with the Helmholtz-Hodge decomposition (HHD). Using contour-guidance significantly ($p<10^{-5}$) decreases the error, reducing the mean error by a factor of 2.2, compared to EVolution. Including the HHD decreases the non-outlier maximum error by a factor of 1.1.

For the simulated cross-contrast prostate experiment, the mean endpoint error on the prostate plus its vicinity of 2 mm before registration was 25.7 mm, see Fig. 3. Using Elastix this became 10.6 mm, and using EVolution this became 5.9 mm. Including contour-guidance, the mean error was reduced by an additional factor of 2.2, to 2.8 mm. After the Helmholtz-Hodge decomposition, the mean error slightly increased to 3.0 mm. When considering a larger area of the prostate and the surrounding 10 mm of tissue, contour-guidance reduced the mean error by a factor of 1.7 to 2.9 mm, indicating that it did not lead to unrealistic deformations outside the guiding contour.

**Fig. 4:**
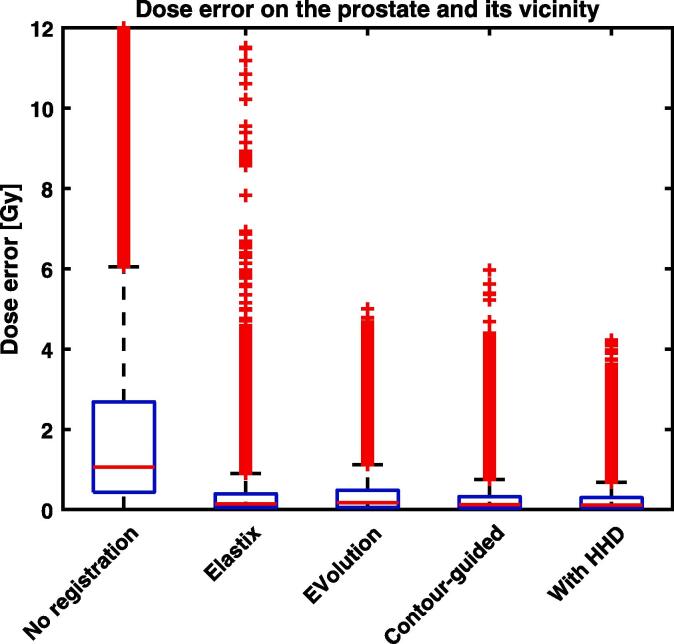
Box plot of the dose error on the prostate and its vicinity of 2 mm for the simulated signal dropout experiment. Shown are the results without registration, using Elastix, using EVolution without contour-guidance, our algorithm with contour guidance, and the algorithm with contour-guidance combined with the Helmholtz-Hodge decomposition (HHD). The maximum error before registration is 33 Gy. Using contour-guidance significantly ($p<10^{-5}$) decreases the error, decreasing the mean, median, 75^*th*^ percentile, and non-outlier maximum with a factor of 1.2, compared to the best non-guided algorithm. The Helmholtz-Hodge decomposition (HHD) decreases the non-outlier maximum error with a factor of 1.1.

For the simulated signal dropout, the mean endpoint error on the prostate plus its vicinity of 2 mm before registration was 4.8 mm, see Figure S7 in the Supplementary Material. This became 1.3 mm after using Elastix or EVolution. Using contour-guidance, the mean endpoint error decreased with an additional factor of 1.5 to 0.9 mm. After performing the Helmholtz-Hodge decomposition, this was further lowered to 0.8 mm. The voxel-by-voxel dose error on the prostate plus vicinity decreased from 2.4 Gy (3.8% of the planned dose) to 0.5 Gy and 0.4 Gy, when using Elastix and EVolution, see Fig. 4. Including contour-guidance decreased the mean dose error with an additional factor of 1.2, to 0.3 Gy. When applying the Helmholtz-Hodge decomposition, the mean dose error slightly decreased further and the maximum error decreased with a factor of 1.2. Also for the dose error on the rectal wall, using contour-guidance on the prostate decreased both the mean and maximum dose errors on this nearby organ-at-risk by a factor of 1.2, compared to the best algorithm without guidance, see Figure S8 in the Supplementary Material. Including the Helmholtz-Hodge decomposition decreased the error with a factor of 1.3.

## 4 Discussion

Using contour-guidance significantly increased contour overlap. Importantly, it significantly decreased the registration error and the dose warping error, compared to the algorithms without contour-guidance. These errors were evaluated on the contour used for guidance and its vicinity, ensuring no errors arise due to over-fitting or boundary inconsistencies. Results confirmed that the proposed algorithm can integrate operator-validated contours into the dose warping and accumulation process by matching deformation vector fields to these contours.

A major contribution of this work is that the proposed solution was specifically designed and validated for low-latency dose accumulation (as well as warping e.g. Hounsfield units) during adaptive radiotherapy workflows. As shown in the work of Willigenburg and colleagues, an MR-guided RT workflow for prostate cancer patients required some degree of post-registration manual correction of the propagated contours in approximately 50% of the cases [37]. This, in turn, invalidated the underlying deformations making them thus unusable for warping quantitative information. Moreover, it is worth taking into account that the study was conducted for intra-fraction adaptations. It is expected that the number of corrections will further increase for inter-fraction cases and/or in areas with higher mobility such as the thorax and the upper abdomen. Prior work in the area of contour-guided image registration has only partially fulfilled the specific requirements for this application. To our knowledge, this is the first study testing a contour-guided registration method on a voxel-by-voxel basis for its registration and dose warping performance. Our method is designed for and validated for multi-modal registrations (as some previous work [9]) while also GPU-accelerated and converging within a few seconds (like [10]). Furthermore, we explicitly incorporated and integrated the contour information and generated a single transformation. Finally, the algorithm was very stable with respect to the (additional) free parameter on a wide range of modalities and anatomies. It was previously indicated that this is a challenge for contour-guided methods [6]. In fact, we used the same parameter configuration for all experiments, in contrast to the algorithms without contour-guidance.

The Helmholtz-Hodge decomposition post-processing step [17], [18], [19] decreased the (non-outlier) range of the Jacobian determinant by about a factor of two and resolved unwanted negative values. For incompressible tissues, like the prostrate on intra-fraction timescales, this brought the Jacobian determinants closer to the simulated benchmark and improved the registration, decreasing the mean and maximum errors.

In many clinical radiotherapy situations where DIR is employed, operator-validated contours are available. Examples include daily plan adaption where contours are propagated to or re-segmented on the anatomy of the day. All adapt-to-shape plan adaption workflows on the MR-linac have validated contours available. With our proposed algorithm, it becomes possible to accumulate the dose for these workflows. An additional application is some inter-fraction registration problems where tissues are not conserved, and a voxel reclassification is needed for registration [38]. We expect that contour-guidance might prove useful in these cases as well, paving the way for additional instances where the warping of quantitative information can be applied. Finally, deep learning may be used for the automatic segmentation of contours to use for guidance. With our method, these contours can be used for warping the dose and CT, for plan comparison, and for treatment response assessment. Additionally, this can improve contour propagation for contours that are not automatically segmented. This may be useful as automatic segmentation can be slow and including additional structures for deep learning segmentation may require retraining. We are currently implementing the algorithm presented here in our clinical workflow to allow these operations. Future work will also focus on validating the algorithm for additional anatomies such as the abdomen.

In conclusion, we introduced a solution for integrating (manually edited) contours in dose warping, matching the deformation vector field with operator-validated contours, and improving the registration performance. The multi-modal algorithm was fast and robust and ensured substantial contour overlap while improving the registration result as well as the warped dose. Importantly, no over-constraining errors were created by the contour-guidance. The algorithm can thus be used to warp doses and other quantitative information in accordance with operator-validated contours, providing a solution for adaptive radiotherapy workflows.

## CRediT authorship contribution statement

**Lando S Bosma:** Conceptualization, Data curation, Methodology, Formal analysis, Investigation, Visualization, Writing - original draft. **Mario Ries:** Conceptualization, Project administration, Funding acquisition, Supervision, Writing - review & editing. **Baudouin Denis de Senneville:** Conceptualization, Software, Writing - review & editing. **Bas W Raaymakers:** Conceptualization, Project administration, Funding acquisition, Supervision, Writing - review & editing. **Cornel Zachiu:** Conceptualization, Data curation, Methodology, Software, Supervision, Validation, Writing - review & editing.

## Declaration of Competing Interest

The authors declare the following financial interests/personal relationships which may be considered as potential competing interests: The collaboration project is co-funded by the PPP Allowance made available by Health-Holland, Top Sector Life Sciences & Health, to stimulate public-private partnerships.

## Appendix A Supplementary data

Supplementary data1

## Footnotes

1 http://bsenneville.free.fr/RealTITracker/

2 https://med.emory.edu/departments/radiation-oncology/research-laboratories/deformable-image-registration/index.html

3 https://learn2reg.grand-challenge.org/Learn2Reg2021/
