# Supplementary material for: Integration of operator-validated contours in deformable image registration for dose accumulation in radiotherapy
Source: Phys Imaging Radiat Oncol. 2023 Aug 20;27:100483. doi: 10.1016/j.phro.2023.100483 (PMC10472292; doi:10.1016/j.phro.2023.100483)
Supplement: Supplementary data1 [file mmc1.pdf]

Supplementary material for *Integration of operator-validated contours in deformable image registration for dose accumulation in radiotherapy*

A. Contour-guidance implementation details

Our proposed algorithm aims to minimize the energy functional

$$\mathcal{E}_{CG} = \int_{\Omega} \mathcal{D}(\vec{u}, I_r, I_m)^2 + \alpha\beta(\nabla M \cdot \vec{u} + M - R)^2 + \alpha\left(||\vec{\nabla}u||_2^2 + ||\vec{\nabla}v||_2^2 + ||\vec{\nabla}w||_2^2\right), \quad (1)$$

where we now write $\mathcal{D}$ for the data fidelity term on the images, M and R for the moving and reference contours, and use u, v, w to denote the three components of the deformation vector field. By applying the Euler-Lagrange equations on a voxel-by-voxel basis, we derive the following system of equations for each voxel:

$$\begin{cases} 2\mathcal{D}\frac{\partial \mathcal{D}}{\partial u} + 2\alpha\beta M_x(\nabla M \cdot \vec{u} + M - R) - 2\alpha\Delta u = 0 \\ 2\mathcal{D}\frac{\partial \mathcal{D}}{\partial v} + 2\alpha\beta M_y(\nabla M \cdot \vec{u} + M - R) - 2\alpha\Delta v = 0 \\ 2\mathcal{D}\frac{\partial \mathcal{D}}{\partial w} + 2\alpha\beta M_z(\nabla M \cdot \vec{u} + M - R) - 2\alpha\Delta w = 0 \end{cases} \quad (2)$$

For a small weight α, the solution of $f(u) = 0$ can be found using an iterative process: $u_{k+1} = u_k - \alpha f(u_k)$ (at convergence $u_{k+1} - u_k$ tends to 0 and thus $f(u_k) = 0$). Applying this to the equations above gives the following fixed-point scheme:

$$\begin{cases} u_{k+1} &= u_k + \Delta u_k - \alpha^{-1}\mathcal{D}\frac{\partial \mathcal{D}}{\partial u} - \beta M_x(\nabla M \cdot \vec{u} + M - R) \\ v_{k+1} &= v_k + \Delta v_k - \alpha^{-1}\mathcal{D}\frac{\partial \mathcal{D}}{\partial v} - \beta M_y(\nabla M \cdot \vec{u} + M - R) \\ w_{k+1} &= w_k + \Delta w_k - \alpha^{-1}\mathcal{D}\frac{\partial \mathcal{D}}{\partial w} - \beta M_z(\nabla M \cdot \vec{u} + M - R) \end{cases} \quad (3)$$

where $k+1$ denotes the new iteration and M_x is the spatial derivative of the reference contour in the x direction.

B. Helmholtz Hodge decomposition derivation and computation details

The Helmholtz-Hodge decomposition can decompose any vector field f in three components: a curl-free component ($\nabla \times f_{cf} = 0$), a divergence-free component ($\nabla \cdot f_{df} = 0$), and a harmonic remainder that is both curl-free and divergence-free ($\nabla \times f_h = 0$, $\nabla \cdot f_h = 0$):

$$f = f_{cf} + f_{df} + f_h. \quad (4)$$

The components are obtained from the equations [1]

$$\begin{aligned} f_{cf} &= \nabla D, \\ f_{df} &= \nabla \times R, \\ f_h &= f - f_{cf} - f_{df}, \end{aligned} \tag{5}$$

where D and R are obtained from the following Poisson equations:

$$\begin{aligned} \Delta D &= \nabla \cdot f, \\ \Delta R &= -\nabla \times f. \end{aligned} \tag{6}$$

We solve these Poisson equations in the spectral domain using the fast Fourier transform [2,3]. For this, consider a (square-integrable) vector field $f(\vec{x})$ and its Fourier transform $\hat{f}(\vec{k})$. In the spectral domain its divergence is $\langle 2\pi i\vec{k}, \hat{f}(\vec{k})\rangle$ and its curl is $2\pi i\vec{k} \times \hat{f}(\vec{k})$, with $\vec{k} = (k_1, k_2, k_3)^\mathsf{T}$. Let us then decompose $\hat{f}(\vec{k})$ in a field that is parallel to $\vec{k}$

$$\hat{f}_{\|} = \langle \hat{f}, \frac{\vec{k}}{\|\vec{k}\|}\rangle \cdot \frac{\vec{k}}{\|\vec{k}\|}, \tag{7}$$

and a part that is perpendicular to $\vec{k}$

$$\hat{f}_{\perp} = -\frac{\vec{k}}{\|\vec{k}\|} \times \left(\frac{\vec{k}}{\|\vec{k}\|} \times \hat{f} \right), \tag{8}$$

such that $\langle 2\pi i\vec{k}, \hat{f}_{\perp}\rangle = 0$ and $2\pi i\vec{k} \times \hat{f}_{\|} = 0$. So, after transforming both parts back into the spatial domain we obtain the curl-free $f_{cf}(\vec{x})$ from $\hat{f}_{\|}(\vec{k})$ and the divergence-free $f_{df}(\vec{x})$ from $\hat{f}_{\perp}(\vec{k})$.

To obtain a divergence-free vector field while preserving as much of the information in f_{cf} as possible, we find the incompressible deformation vector field using

$$f_i = f_{df} + (1 + a) f_h + f_t \tag{9}$$

where a is a free parameter and f_t is a translation field given by three free parameters $b_{1,2,3}$. These free parameters are found by minimizing [1]

$$\min_{a, b_{1,2,3}} \|a \cdot f_h + f_t - f_{cf}\|^2, \tag{10}$$

thus preserving as much of the information in f_{cf} as possible. The result is divergence-free. The decomposition is implemented in MATLAB (version 2019a, MathWorks Inc, Natick, USA).

C. Evaluation criteria

The Jacobian determinant indicates the amount of volumetric expansion (if larger than 1) or contraction (smaller than 1) given by the estimated transformation. Values below

0 indicate tissue folding and are generally unwanted. The Jacobian determinant is expected to be close to 1 for watery tissues. The Jacobian determinant was evaluated on the prostate contour; the liver, spleen, and kidney contours; and the body minus the lung contours, for the different experiments. The same regions were used for the Helmholtz-Hodge decomposition.

For the simulated signal dropout data, we evaluated the dose warping performance by comparing the planned dose distribution warped using both the simulated and estimated deformation vector fields. The dose error is the absolute difference between the two. The energy per mass transfer (EMT) method was used to warp the dose [4, 5].

For the prostate anatomies, the prostate contour was used for guidance and for the HHD. For the abdomen anatomies, the liver, spleen, and kidney contours were used for guidance and the HHD. For the thorax anatomies, the lungs were used for guidance and the body excluding the lungs was used for the HHD. The contours used for guidance were also used for the evaluation using mean distance to agreement and Hausdorff distance.

D. Acquisition details

For the large and complex deformations of the prostate anatomy, the pretreatment images were acquired using a 3D T2 transversal spin echo (repetition time 2500 ms, echo time 357 ms, flip angle 90°, field strength 3.0 T). The daily (pre-beam) images were acquired on the 1.5T MR-Linac Unity sytem (Elekta AB, Stockholm, Sweden) using a spin-echo sequence (repetition time 1535 ms, 278 ms, flip angle 90°, field strength 1.5 T). The study was conducted in agreement with the required standards and regulatory requirements. Ethical approval was provided by the Ethics Board of the University Medical Center Utrecht.

The cross-contrast experiment used a set of DIXON images (repetition time 3.9 ms, echo time 1.2/2.5 ms, flip angle 10°, field strength 3.0 T). The voxel dimensions are $(1.9 \times 1.9 \times 2.3)$ mm^3.

E. Elastix parameters

For the abdominal CT to MR registrations, we use the parameter set Par0023 from the 'Model Zoo', as this was optimized for CT to MR registrations. For the thoracic 4DCT, we use the set Par0008 that was previously optimized for thoracic 4DCT registrations. For the other experiments, we use the 'default' deformable parameter set, with the number of iterations increased to 1000.

References

[1] Fu T, Fan J, Liu D, Song H, Zhang C, Ai D, et al. Divergence-Free Fitting-Based Incompressible Deformation Quantification of Liver. IEEE Journal of Biomedical and Health Informatics. 2020;25(3):720-36.

Table S1: Hausdorff distance in mm for the different experiments. Shown are the results before registration, with Elastix, EVolution without contour-guidance, EVolution with contour-guidance, and EVolution with contour-guidance and the Helmholtz-Hodge decomposition (HHD). For the large complex deformations and multi-modal experiments, we show the mean values. Contour-guidance reduces the Hausdorff distance by a factor of 1.9 on average (range 1.1-3.3), compared to the best algorithm without guidance. After the HHD the contour overlap is still considerably improved.

Experiment	No DIR	Elastix	EVolution	Contour-guided	With HHD
Large complex deformations prostate	26.1 (16.6)	12.8 (6.6)	11.5 (6.4)	7.4 (7.0)	7.6 (6.9)
Large complex deformations thorax	31.2 (17.7)	17.7 (11.2)	22.4 (12.5)	15.7 (12.3)	14.3 (11.1)
Signal dropout prostate	15.6	10.3	9.9	3.0	4.0
Signal dropout simulation prostate	7.5	4.1	5.7	3.0	3.0
Multi-modal abdomen	48.7 (21.9)	33.1 (28.1)	31.4 (21.6)	17.8 (17.7)	24.8 (16.8)
Dixon cross-contrast simulation prostate	26.9	9.0	5.7	2.4	4.1

Table S2: Mean distance to agreement in mm evaluated on the different organs of the multi-modal MR-to-CT abdominal experiment. Shown are the mean (standard deviations) results before registration, with Elastix, EVolution without contour-guidance, EVolution with contour-guidance, and EVolution with contour-guidance and the Helmholtz-Hodge decomposition (HHD).

Organ	No DIR	Elastix	EVolution	Contour-guided	With HHD
All organs	13.3 (12.0)	6.0 (12.5)	4.6 (9.3)	0.7 (2.4)	1.8 (2.9)
Liver	9.6 (7.3)	5.1 (7.5)	3.6 (5.7)	0.2 (0.4)	0.8 (1.0)
Spleen	14.4 (14.2)	7.7 (14.3)	5.7 (8.6)	0.3 (0.4)	1.8 (2.3)
Right kidney	16.0 (15.7)	8.3 (18.8)	6.1 (15.2)	1.8 (4.7)	2.9 (4.9)
Left kidney	13.3 (9.7)	2.1 (2.9)	2.2 (3.1)	0.2 (0.1)	1.5 (1.1)

Table S3: Mean computation times in seconds for different anatomies, given as mean (standard deviation) over the number of registrations (n). Shown are the registration times for Elastix, EVolution without contour-guidance, and EVolution with contour-guidance. The computation time of the Helmholtz-Hodge decomposition (HHD) is also shown.

Anatomy	Elastix	EVolution	Contour-guided	HHD
Prostate MR-MR (20)	50.8 (0.9)	3.6 (0.4)	3.1 (0.6)	1.8 (0.1)
Abdomen CT-MR (8)	407 (12.5)	5.3 (0.3)	2.8 (0.2)	1.9 (0.3)
Thorax CT-CT (20)	7.5 (7.7)	4.9 (1.0)	9.8 (3.0)	22.4 (1.5)

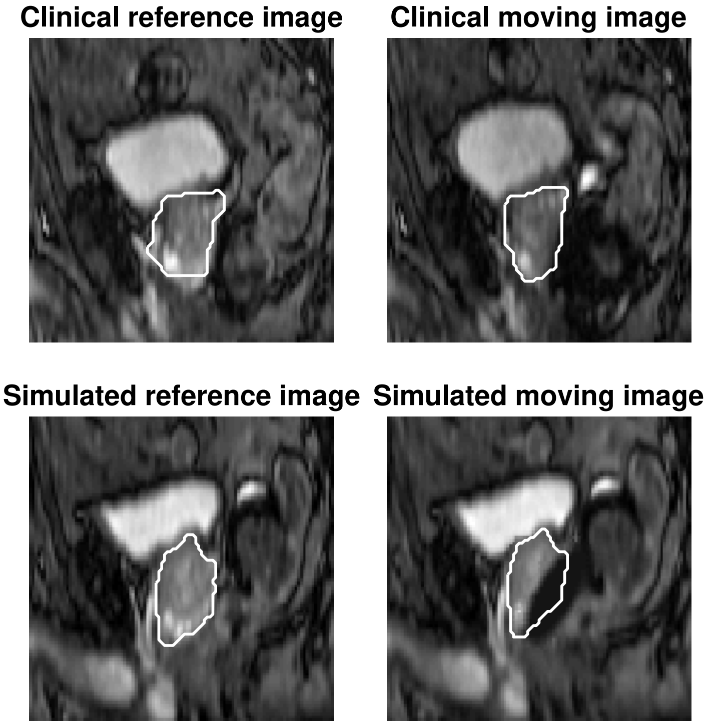

Figure S1: Sagittal slice for both the clinical example of a signal dropout due to a gas bubble in the rectum and the simulated signal dropout. The prostate contour used for guidance is indicated in white.

Table S4: Jacobian determinant non-outlier ranges for the different experiments. Outliers are determined by the 25th (respectively 75th) percentile minus (plus) 1.5 times the range between the 25th and 75th. The ranges are determined on the prostate; liver, spleen, and both kidneys; full body except for the lungs. For experiments with multiple registrations, we show the median. By employing the Helmholtz-Hodge decomposition (HHD), the range decreases by a factor of 2.0 on average (range: 1.7-2.6).

Experiment	Benchmark	Elastix	EVolution	Contour-guided	With HHD
Large complex deformations prostate		(0.5, 1.4)	(0.6, 1.3)	(0.4, 1.4)	(0.7, 1.3)
Large complex deformations thorax		(0.6, 1.3)	(0.3, 1.5)	(0.7, 1.2)	(0.9, 1.2)
Signal dropout prostate		(-0.1, 1.5)	(0.7, 1.2)	(0.3, 1.6)	(0.7, 1.3)
Signal dropout simulation prostate	(0.9, 1.1)	(0.6, 1.3)	(0.6, 1.5)	(0.5, 1.5)	(0.8, 1.3)
Multi-modal abdomen		(0.3, 1.6)	(0.0, 2.0)	(0.0, 1.9)	(0.5, 1.5)
Dixon cross contrast simulation prostate	(1.0, 1.4)	(-0.2, 2.5)	(0.9, 2.2)	(0.6, 2.4)	(0.7, 1.4)

[2] Reich W, Hlawitschka M, Scheuermann G. Decomposition of vector fields beyond problems of first order and their applications. In: Topological Methods in Data Analysis and Visualization. Springer; 2017. p. 205-19.

[3] Littlejohn R. The classical electromagnetic field hamiltonian (Lecture Notes). UC Berkeley Physics, University of California; 2021. Available from: `https://bohr.physics.berkeley.`

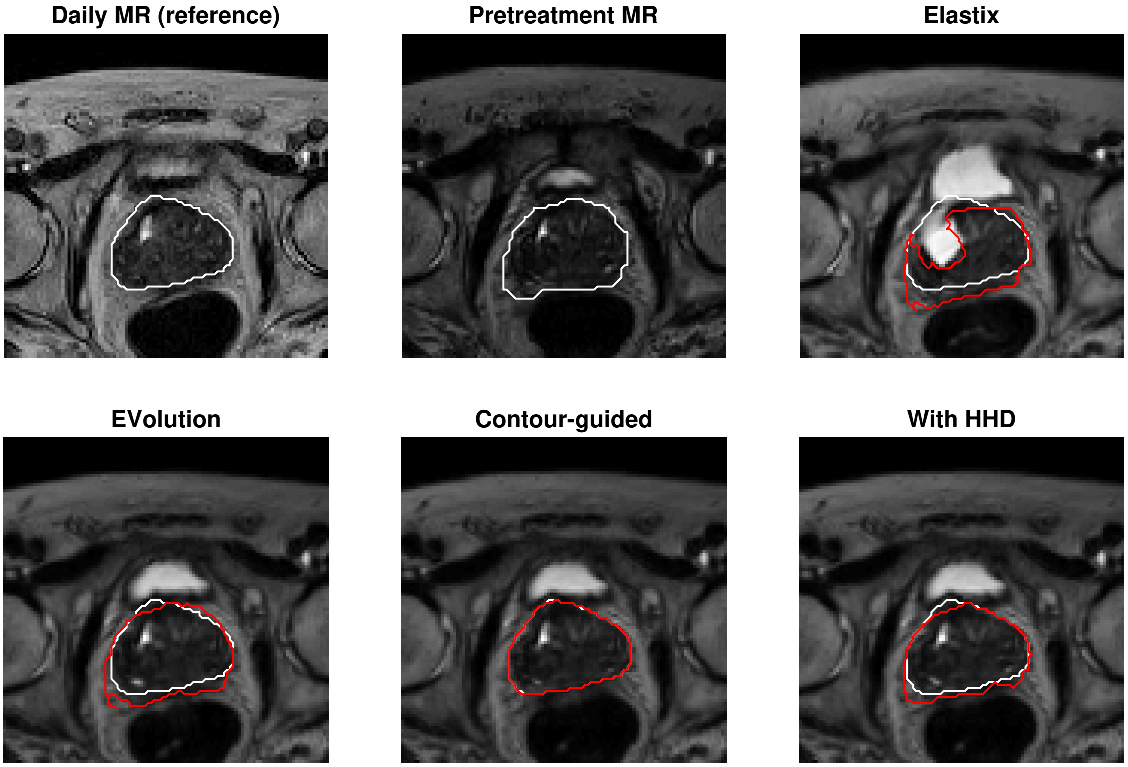

Figure S2: An example case of the MR-to-MR registrations for large complex deformations on the prostate anatomy. A transversal slice of the daily MR and pretreatment MR is shown, as well as the pretreatment MR registered to the daily MR using Elastix, the original EVolution, our proposed contour-guided algorithm, and this contour-guided algorithm with the Helmholtz-Hodge decomposition (HHD) on the prostate. The white lines denote the contours used for guidance and the red lines denote the registered contours. The improved prostate overlap when using contour-guidance is clearly visible.

`edu/classes/221/2122/221.html`.

[4] Li HS, Zhong H, Kim J, Glide-Hurst C, Gulam M, Nurushev TS, et al. Direct dose mapping versus energy/mass transfer mapping for 4D dose accumulation: fundamental differences and dosimetric consequences. Physics in Medicine & Biology. 2013;59(1):173.

[5] Bosma L, Zachiu C, Ries MG, de Senneville BD, Raaymakers BW. Quantitative investigation of dose accumulation errors from intra-fraction motion in MRgRT for prostate cancer. Physics in Medicine & Biology. 2021.

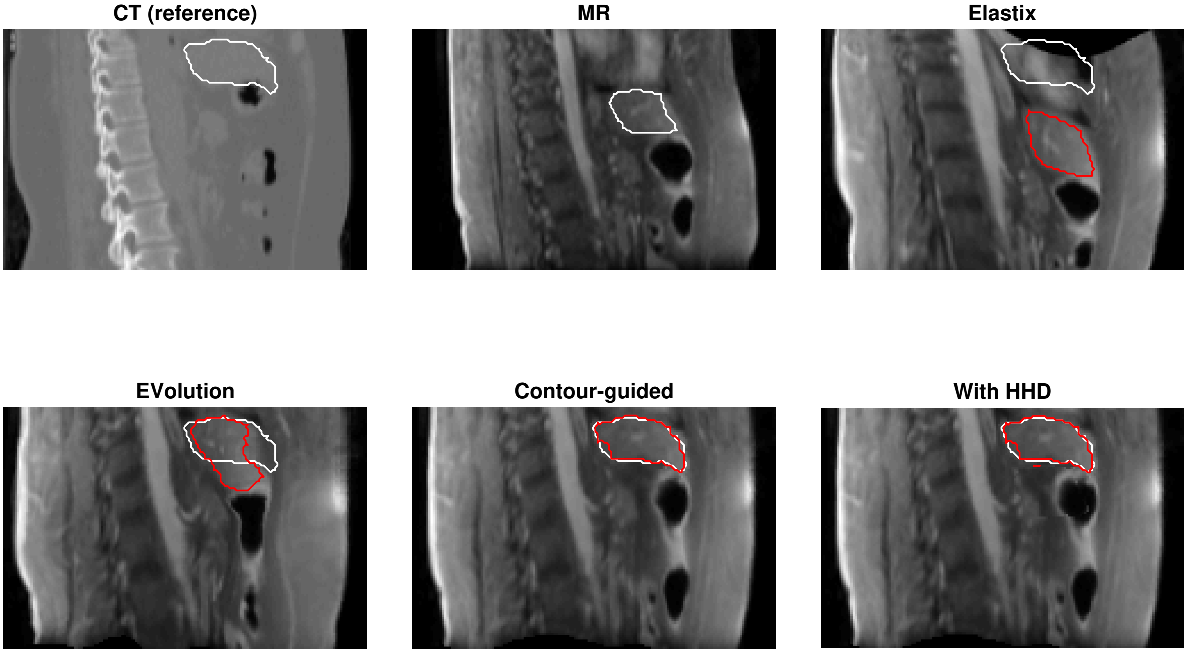

Figure S3: An example case for the cross-contrast abdominal MR-to-CT registrations. A sagittal slice of the CT and MR images is shown, as well as the MR registered to the CT using Elastix, the original EVolution, our proposed contour-guided algorithm, and this contour-guided algorithm with the Helmholtz-Hodge decomposition (HHD) on the abdominal organs. The contours used for guidance are shown in white and the registered contours are shown in red. The improved contour and image alignment when using contour-guidance is clearly visible.

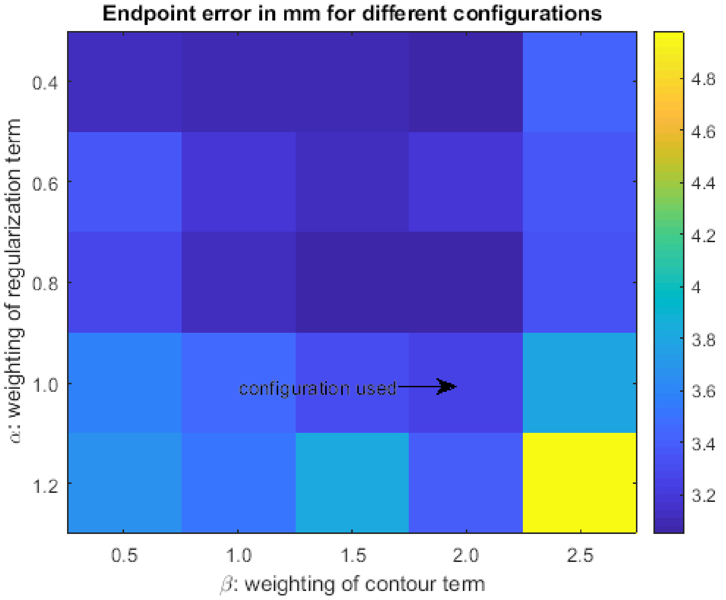

Figure S4: Mean endpoint error in mm on the prostate plus vicinity for the cross-contrast MR experiment for different configurations of the contour-guidance algorithm. The variation is relatively small with the configuration used giving 3.3 mm and the optimal configuration 3.1 mm.

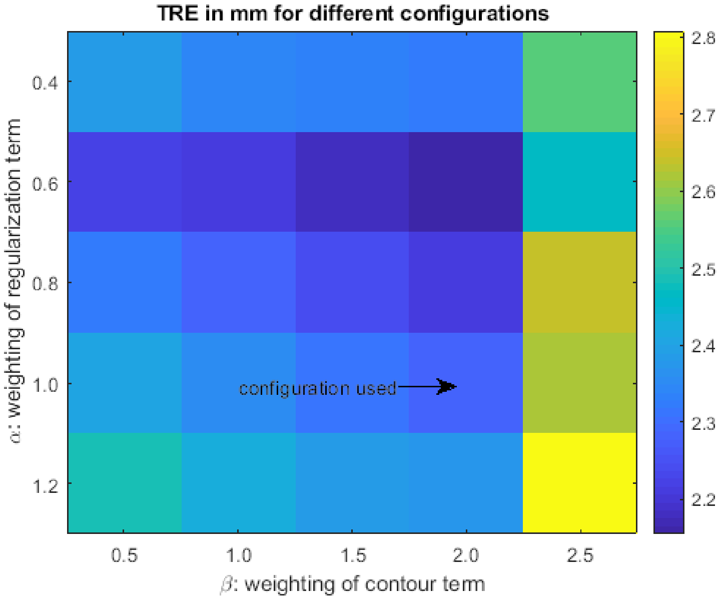

Figure S5: Mean TRE in mm for the thorax CT-to-CT experiment for different configurations of the contour-guidance algorithm. The variation is relatively small with the configuration used giving 2.3 mm and the optimal configuration 2.2 mm.

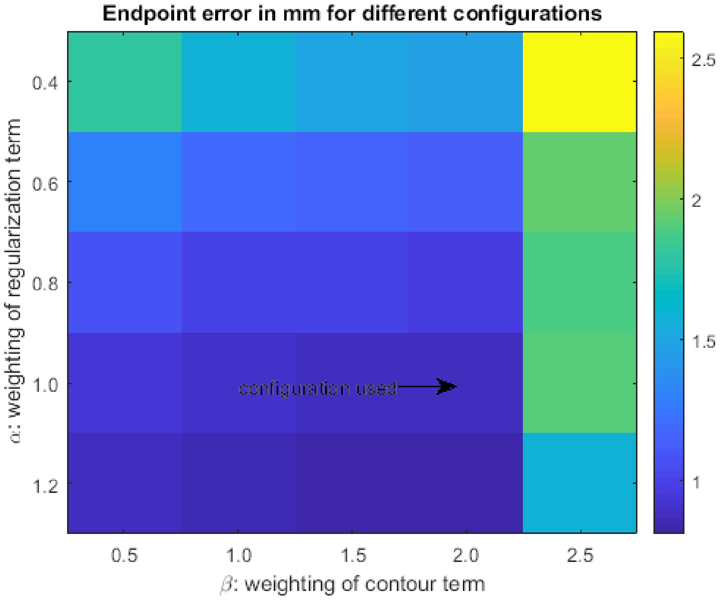

Figure S6: Mean endpoint error in mm on the prostate plus vicinity for the simulated signal dropout MR-to-MR experiment for different configurations of the contour-guidance algorithm. The variation is relatively small with the configuration used giving 0.9 mm and the optimal configuration 0.8 mm.

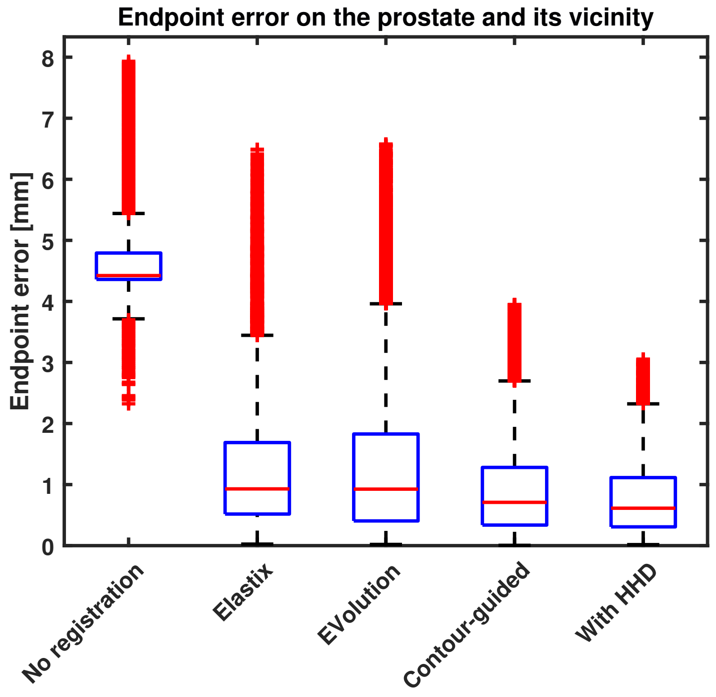

Figure S7: Box plot of the voxel-by-voxel endpoint error on the prostate and its 2 mm vicinity for the simulated signal dropout. Shown are the results without registration, using Elastix, using EVolution without contour-guidance, our algorithm with contour guidance, and the algorithm with contour-guidance combined with the Helmholtz-Hodge decomposition (HHD). Using contour-guidance decreases the mean endpoint error with a factor of 1.6, to 0.8 mm. The Helmholtz-Hodge decomposition decreases the maximum error by a factor of 1.3.

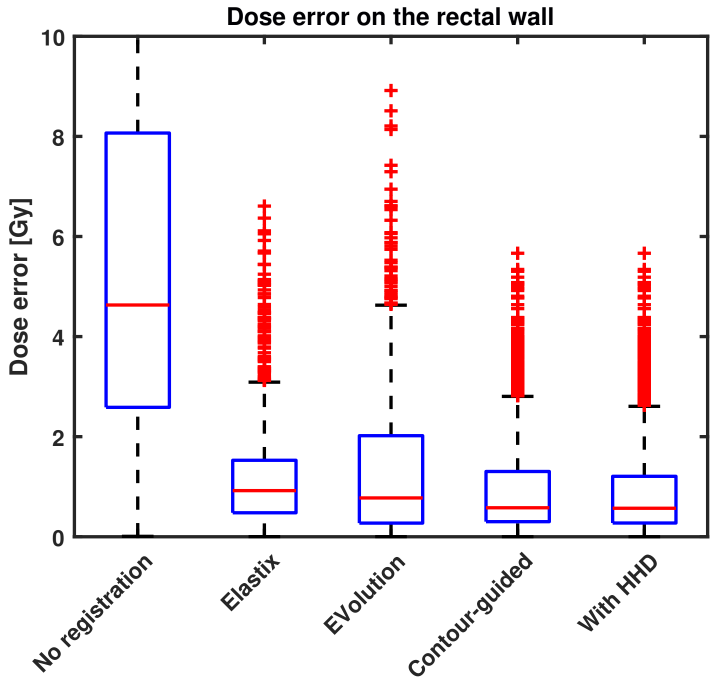

Figure S8: Box plot of the dose error on the rectal wall for the simulated signal dropout experiment. Shown are the results without registration, using Elastix, using EVolution without contour-guidance, our algorithm with contour guidance on the prostate contour, and the algorithm with contour-guidance combined with the Helmholtz-Hodge decomposition (HHD). The maximum error before registration is 22.4 Gy. Using contour-guidance significantly ($p < 10^{-9}$) decreases the error, decreasing both the mean and maximum errors with a factor of 1.2, compared to the best algorithm without contour-guidance. The Helmholtz-Hodge decomposition (HHD) decreases the mean and maximum errors with a factor of 1.3 and 1.2.
